# Abrupt Transition of Nanothermite Reactivity: The Roles of Loading Density, Microstructure and Ingredients

**DOI:** 10.3390/molecules30204101

**Published:** 2025-10-15

**Authors:** Chengbo Ru, Yanchun Zhang, Aoyang Yu, Lihong Chen, Hongxing Wang, Hongguo Zhang, Yiming Shan, Yi Jin

**Affiliations:** 1 College of Forensic Science, Criminal Investigation Police University of China, Shenyang 110035, China; ruchengbo@163.com (C.R.);; 2 Key Laboratory of Impression Evidence Examination and Identification Technology, Ministry of Public Security, Shenyang 110035, China; 3 Graduate School, Shenyang Ligong University, Shenyang 110159, China

**Keywords:** nanothermites, energetic additives, loading density, abrupt reactivity transition, heat transfer

## Abstract

Nanothermites are widely applied as specific power sources for microscale initiators and pyrotechnics. Increasing the charge density enhances energy storage within a confined combustion chamber, but it also alters the reaction kinetics. To systemically explore this phenomenon, the combustion and pressurization characteristics of electrosprayed nanothermite-based hybrid energetic materials (THEMs) with different metallic oxides (Fe_2_O_3_, CuO, and Bi_2_O_3_) and various energetic additives (nitrocellulose (NC), octogen (HMX), ammonium perchlorate (AP), and hexanitrohexaazaisowurtzitane (CL-20)) across various loading densities were tested. The results showed that increasing the loading density decreased the porosity of the loaded nanothermites and then rapidly decreased the convective heat transfer efficiency during the combustion propagation process. When the loading density exceeded a critical value, a dramatic decrease in the peak pressure, several orders-of-magnitude decrease in the pressurization rate, and an order-of-magnitude increase in the combustion duration occurred. Due to the dual effects of the porous microstructure on heat and mass transfer, the critical density of both the electrosprayed Al/CuO/NC/CL-20 composites and their physically mixed counterparts is between 37.9 and 43.9% theoretical maximum density (TMD). Because of the different synergistic catalytic effects, the fast reactivity at the high-loading-density maintaining capacity of the applied additives was AP > HMX ≈ CL-20 > NC. Owing to their intrinsic properties of low ignition temperature and high gas yield, the Bi_2_O_3_-THEMs could maintain high-speed reactivity even at 59.7% TMD. These results provide valuable insights into the rational design and tailoring of the reactivity of nanothermites for specific applications.

## 1. Introduction

As a specialized subclass of pyrotechnics, nanothermites consisting of fuel nanoparticles (Al, B, and Si) and oxidizer nanoparticles (metallic oxides, sulfates, perchlorates, iodates, and fluorine-containing polymers) are of considerable interest to researchers [1]. Nanothermites can serve not only as power sources for primers, solid propellant microthrusters, drug delivery systems, ignition chains, safe and arming devices, and circuit breakers but also as biocidal agents [2,3]. Unlike organic monomolecular energetic materials, pyrotechnics react via combustion or deflagration, which is governed mainly by the heat and mass transport efficiency between reactants. For nanothermites, component dispersion homogeneity, severe nanoparticle reactive sintering [4] and low gas production play crucial roles in limiting output performance.

In the last two decades, researchers have focused primarily on resolving these issues. Architectures, such as core–shell [5,6], dense laminate [7], porous foam [8], assembled microparticle [9,10], nanofibrous [5] and other architectures [11], have been constructed to enhance component distribution uniformity and enlarge the fuel–oxide contact area, thus improving heat and mass transport efficiency. Reactive sintering, where fuel nanoparticles rapidly lose their nanostructures at high heating rates, severely hinders the completion of thermite reactions. Hence, H. Wang et al. [10] introduced nitrocellulose (NC) into nanothermites via electrospray to avoid severe sintering of the Al nanoparticles with the aid of NC gaseous decomposition products. Nanothermites also can enhance the combustion performance of NC by promoting the cleavage of ONO_2_ bonds within NC and the adsorption of NO_x_ molecules [12]. Modifying the reaction process of nanothermites by doping additives such as high explosives [13,14], gas agents [15], strong oxidizing agents [16,17], and carbon materials [18] is also a powerful approach to complement the combustion process. Y. Zhu et al. [13] integrated high explosive hexanitrohexaazaisowurtzitane (CL-20) with core/shell CuO/Al nanowires, resulting in more violent combustion propagation accompanied by a crisp sound. Y. Hu et al. [17] applied ammonium perchlorate (AP), which can provide enough oxygen, as additives for different nanothermite pairings, such as Al/CuO, Al/MoO_3_, Al/Bi_2_O_3_, Al/Fe_2_O_3_, Al/NiO and Al/MgO, resulting in shorter ignition delay times, greater heat release and higher flame temperatures.

Owing to these efforts, nanothermites possessing high energy density and smaller critical diameters for self-sustaining combustion can fulfill the specific power source requirements of microscale initiators and pyrotechnic devices. However, in practical applications, the charge-loading wells in these microsystems are extremely small, as their feature sizes continue to shrink for space saving. Therefore, nanothermite powders often need to be pressed into high-density grains to maximize the charge loading. However, increasing the loading density of nanothermites significantly alters heat and mass transfer during combustion propagation, leading to a decline in output performance, which is entirely different from that of organic high-explosives (such as trinitrotoluene and CL-20). As reported in previous works, above a critical loading density, an abrupt decrease in reactivity occurs, such as combustion propagation speed [19,20,21,22], peak thrust [23,24,25] or pressurization characteristics [26]. For example, abrupt propulsion performance transitions of Al/CuO [24] and Al/Bi_2_O_3_/NC(2.5%) [23] nanothermites occurred at 44.4% TMD and 52.5% TMD, respectively. Their impulse durations increased by more than an order of magnitude, and the values of thrust correspondingly decreased by more than an order of magnitude. Notably, their specific impulses (impulses per weight) remained relatively constant across loading densities, indicating that the total output energy was unaffected. In the case of Al/MoO_3_ nanothermites [21], the combustion propagation speed decreased three orders of magnitude across a 6.5 to 73% TMD.

Researchers have attempted to elucidate the dominant heat transfer mechanisms underlying this phenomenon [7,27,28], i.e., an abrupt transition from convective to conductive. From a microscopic perspective, voids between reactants inevitably persist in loaded energetic materials, and their quantity plays a decisive role in determining the heat transfer dominant mode during combustion propagation. At low loading densities, convection through voids is widely accepted to dominate heat transfer. Calculations by E. Garth et al. [28] demonstrated that the convection of gaseous products and condensed phase species are primary contributors to high-speed combustion propagation in the loosely packed charge (6% TMD) of Al/CuO nanothermites, whereas the contributions of conduction and radiation are insufficient. In addition, D. Kline [27] added 5~25 wt% CuO nanoparticles to supply additional condensing metal vapors, guaranteeing stable combustion in printed Al/I_2_O_5_ sticks. Increasing the loading density reduces the number of voids within the loaded charges, thus increasing the thermal conductivity of unreacted materials [29,30], but shuts down channels for the convection of gaseous and condensed products [31]. Consequently, thermal conduction becomes the dominant heat transfer mode at high loading densities, leading to a reduced combustion propagation efficiency. Notably, T. Wu et al. [7] reported that trapped air within ~20 vol% micropores accelerated the burn rate of fully dense Al/CuO nanolaminates (dominated by thermal conduction) by 18%.

For specific applications, it is crucial to understand and regulate the reactivity of nanothermites. For example, when used as a substitute for primary explosives [32,33,34] or for generating large instantaneous thrusts in the case of solid propellant microthrusters for emergency obstacle avoidance, loosely packed nanothermites with high reaction rates are preferred. Conversely, the reaction rates of nanothermites need to be slowed down to generate a small-scale thrust with prolonged durations to achieve precise attitude adjustments [35] or to output a sufficient high temperature for long durations to melt the circuit when used in self-destructing energetic chips [36]. Hence, we systematically investigated the abrupt transition behavior of various nanothermite pairs and explored the underlying regulatory mechanism. In this work, metallic oxides and aluminum were assembled into the matrix of NC and energetic additives to form microparticles by electrospray. CuO, Fe_2_O_3_ and Bi_2_O_3_ were selected as metallic oxides because of their distinct chemical properties, which lead to different reaction mechanisms and performances [37,38].

It should be noted that nanothermites possess a significant limitation. Their reaction products primarily consist of metals and metal oxides with high boiling points and molecular weights, resulting in low gas generation. Incorporating gas agents, such as propellant components like NC and AP, as well as high explosives yielding substantial gaseous products (as shown in Table S1) can moderate this problem. These agents not only generate large volumes of low-molecular-weight gaseous products leading to enhanced convective heat transfer efficiency with improved local high pressure but also exhibit synergistic catalytic interactions with nanoparticles of aluminum and metal oxides [39], further increasing the completeness of the reaction. NC was chosen to improve the viscosity of the precursors, preventing nanoparticle sedimentation during the electrospray process. In addition, the decomposition products of NC can prevent rapid sintering of Al and metallic oxide nanoparticles, resulting in an enhanced combustion performance [10]. As discussed above, although numerous studies have been conducted, no systematic comparison of the effects of various gas-producing agents has been reported. Hence, NC, octogen (HMX), CL-20 and AP were selected as energetic additives to compare the effect of energetic additive species on the reactivity of nanothermites. Previous studies have confirmed that electrosprayed microparticles exhibit superior combustion performance owing to their unique microstructures and the presence of gas agents [40,41]. The combustion behaviors and pressurization characteristics of the prepared nanothermites loaded at various densities were subsequently tested to investigate the roles of loading density, microstructure, additive species and metallic oxides in regulating abrupt transition behaviors of reactivity.

## 2. Results

### 2.1. Morphology and Component Distribution

**Figure 1:**
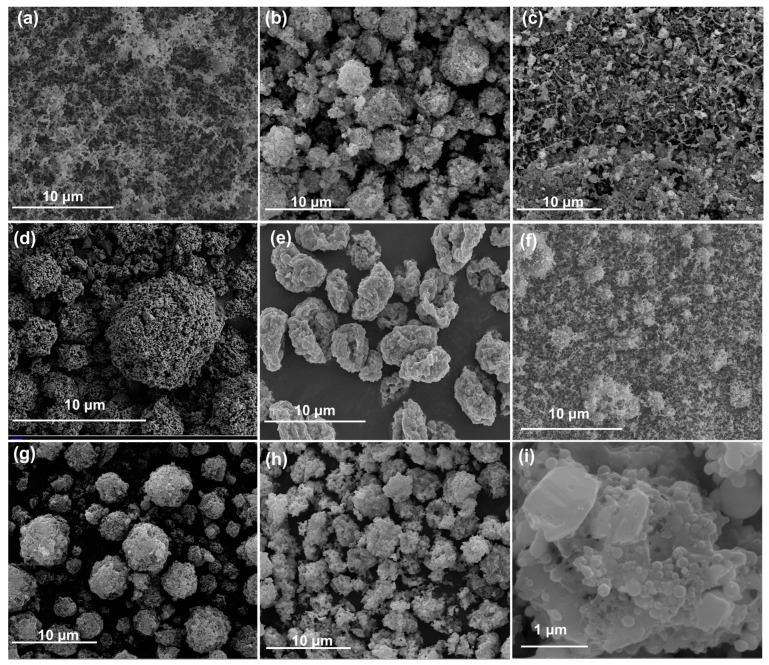
Morphologies of the prepared THEMs. SEM images of (**a**) Al/CuO, (**b**) Al/CuO/NC, (**c**) Al/CuO/NC/AP, (**d**) Al/CuO/NC/HMX, (**e**) Al/CuO/NC/CL-20, (**f**) Al/CuO/NC/CL-20 (UM), (**g**) Al/Fe_2_O_3_/NC/CL-20, (**h**) Al/Bi_2_O_3_/NC/CL-20, and (**i**) enlarged Al/Bi_2_O_3_/NC/CL-20.

Figure 1 and Figure S1 show SEM images and EDS mapping of the as-prepared THEMs. The ultrasonic-mixed nanothermites of Al/CuO appeared as uniform loose powders with no obvious agglomerates, as shown in Figure 1a. With the aid of an external electric field, electrospray can assemble nanothermites and additives into microparticles with feature sizes ranging from 1.8 to 8.1 μm (Figure 1b–h), except for Al/CuO/NC/AP. The Al and metallic oxide nanoparticles dispersed evenly and were encapsulated in the matrix of the NC and high explosive materials (CL-20 or HMX), which underwent precipitation and recrystallization, as shown in Figure 1c. Porous structures on the surface and inside (as reported by Wang [42]) of the as-sprayed microparticles naturally formed due to fast evaporation and the existence of a solid concentration gradient from the surface toward the droplet center. The intrinsic nature of the AP increases the electrical conductivity of the THEM precursor and then disturbs the steady cone–jet mode into the spindle mode, generating larger droplets and fragments under identical operating conditions. Therefore, no obvious encapsulated microparticles of Al/CuO/NC/AP or the matrix of energetic additives were observed, as shown in Figure S1.

### 2.2. Combustion Behaviors of the Assembled Nanothermites

**Figure 2:**
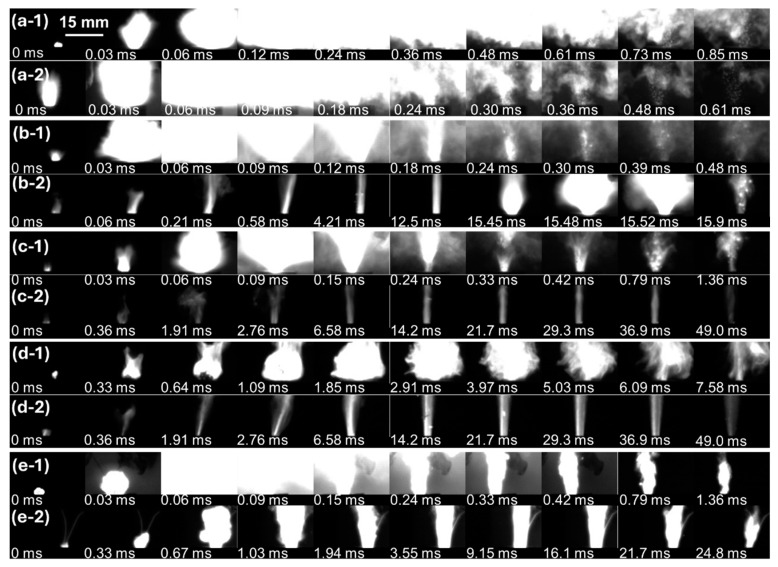
Combustion behaviors of the assembled THEMs at various densities recorded by a high-speed camera. Al/CuO at (**a-1**) 28.5% TMD and (**a-2**) 47.5% TMD; Al/CuO/NC/AP at (**b-1**) 28.9% TMD and (**b-2**) 76.2% TMD; Al/CuO/NC/HMX at (**c-1**) 29.1% TMD and (**c-2**) 49.6% TMD; Al/Fe_2_O_3_/NC/CL-20 at (**d-1**) 19.4% TMD and (**d-2**) 47.7% TMD; and Al/Bi_2_O_3_/NC/CL-20 at (**e-1**) 29.7% TMD and (**e-2**) 72.5% TMD. The applied aperture and exposure time are F32 and 15 μs in (**a**–**d**) and F2.8 and 15 μs in (**e**), respectively.

In previous works, increasing the loading density of nanothermites above a certain value caused an abrupt transition in reactivity. Similarly, the combustion behaviors of the THEMs but not the Al/CuO (UM) composite at various loading densities revealed two distinct phenomena, rapid combustion (low-density) and slow combustion (high-density), as shown in Figure 2 and Figure S2. In the case of Al/CuO (UM), there was no visible difference in the brightness or dimensions of the flames at various loading densities, which was consistent with its pressurization performance. The combustion processes of Al/CuO at 28.5% TMD and 47.5% TMD were similar: masses of materials were ejected from the charge well rapidly within ~0.4 ms, and the combustion durations were both below 1.0 ms. The ejection of the loaded materials may be caused by the combustion wave front propagating at supersonic speed reaching the bottom of the charge well and then reflecting off before hot products move out [24]. After the addition of the gas agents of NC and energetic additives, distinct combustion behaviors appeared. At low density (~30% TMD), violent and luminous flames could be observed within ~0.10 ms, and the combustion durations were all on the order of 1 ms. At high density, the THEMs combusted in the form of steady end-burning with darker and localized plumes, which is similar to the combustion behavior of solid propellants loaded in rocket motors. The reaction propagation rate decreased dramatically, leading to an order of magnitude increase in combustion duration (~50 vs. ~1.5 ms). Notably, at the very end (15.6 ms) of combustion of the Al/CuO/NC/AP at 76.2% TMD, a violent ejected flare appeared (Figure 2(b-2)) and induced a jump in real-time pressure and optical emission. The loaded Al/CuO/NC/AP pellets generated higher pressure in a shorter time than did the other Al/CuO-THEMs across all loading densities. Therefore, a more powerful combustion wave may lead to a violent ejection, which is ubiquitous at low loading densities. The distinct combustion behaviors of the Fe_2_O_3_-THEMs and Bi_2_O_3_-THEMs at low and high densities were similar to those of Al/CuO/NC/CL-20. Owing to the low gaseous product yield capacity and poor reactivity of Al/Fe_2_O_3_, the combustion of Al/Fe_2_O_3_/NC/CL-20 lasted as long as 7.58 ms. The low Tammann temperature of Bi_2_O_3_ and low vaporization point of Bi led to much more violent combustion of the Bi_2_O_3_-THEMs, during which the main combustion was complete in 0.12 ms. At high density, both the Fe_2_O_3_-THEMs and Bi_2_O_3_-THEMs combusted in an end-burning manner with steady and rayless plumes, which lasted more than 25 ms.

### 2.3. Pressurization Characteristics of the Al/CuO/NC/Additives

**Figure 3:**
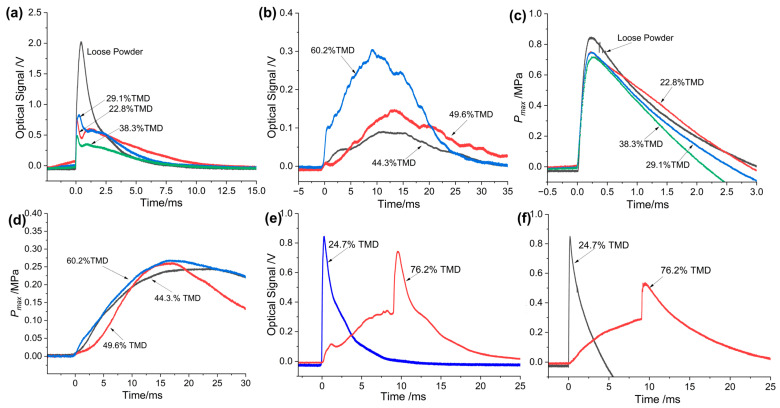
Optical emission profiles of Al/CuO/NC/HMX at (**a**) low loading densites and (**b**) high loading densities. Pressure profiles of Al/CuO/NC/HMX at (**c**) low loading densites and (**d**) high loading densities. (**e**) Optical emission and (**f**) pressure profiles of Al/CuO/NC/AP at 24.7%TMD and 76.2%TMD.

As expected, the pressurization characteristics of the as-prepared THEMs loaded at low and high densities were quite different, which is an inevitable consequence of combustion behavior. For example, the pressure and optical emission plots of Al/CuO/NC/HMX at the 22.8% to 60.2% TMD range measured by a combustion cell are shown in Figure 3a–d. Obviously, the pressure and optical profiles at low densities (22.8 to 38.3% TMD) were much greater, with a more rapidly rising slope than those at high densities (44.3 to 60.2% TMD). As the loading density increased from 38.3 to 44.3% TMD, the pressure rising time (the time it takes to reach the maximum value) increased by more than one order of magnitude; in other words, an abrupt transition in the pressurization performance of the Al/CuO/NC/HMX pellets occurred. Figure 3e,f shows the pressure and optical emission profiles of Al/CuO/NC/AP. A jump in pressure and optical emission clearly occurred at the very end (15.6 ms) of the combustion of the Al/CuO/NC/AP composite at 76.2% TMD, as discussed above.

**Figure 4:**
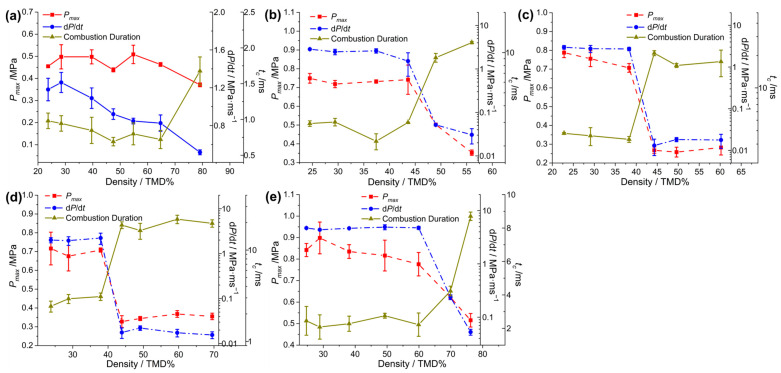
Measured pressurization characteristics of (**a**) Al/CuO, (**b**) Al/CuO/NC, (**c**) Al/CuO/NC/HMX, (**d**) Al/CuO/NC/CL-20, and (**e**) Al/CuO/NC/AP as a function of %TMD.

For a more intuitive comparison, the peak pressure (*P_max_*), pressurization rate (d*P*/d*t*) and combustion duration (*t_c_*) of the Al/CuO-THEMs were extracted and plotted, as shown in Figure 4. Then, the abrupt reactivity transition of the THEMs could be deduced from the pressurization characteristics. For Al/CuO, the overall tendency of d*P*/d*t* decreased with increasing loading density, decreasing from 1.26 MPa/ms (28.5% TMD) to 0.52 MPa/ms (79.1% TMD). In the range of 23.7 to 64.7% TMD, the values of *P_max_* and *t_c_* fluctuated with no clear trend; however, a significant change appeared (0.46 MPa vs. 0.37 MPa and 0.72 ms vs. 1.67 ms, respectively) when the loading density increased from 64.7 to 79.1% TMD. The reactivity of Al/CuO at 28.5% TMD is likely superior to that at 23.7% TMD, and this trend is similar to the results of Al/CuO-nanorods measured in the range of 23~30% TMD by R. Thiruvengadathan [43], which can be attribute to the increased heat transfer by conduction.

After the introduction of NC and energetic additives, the abrupt transitions in the reactivity of the CuO-THEMs became clear and appeared in different loading density regimes. When the combustion crossed from the low-density regime to the high-density regime, d*P*/d*t* decreased by orders of magnitude, the value of *P_max_* decreased by more than half, and, correspondingly, *t_c_* increased several times, as shown in Table 1. For Al/CuO/NC, a sharp decrease in reactivity was observed in the 43.8 to 49.0% TMD range, in which d*P*/d*t* decreased by more than two orders of magnitude (1.51 vs. 0.05 MPa/ms), *t_c_* increased threefold (3.0 vs. 9.2 ms), and *P_max_* decreased from 0.74 to 0.50 MPa. However, the abrupt transition zones of Al/CuO/NC/CL-20 and Al/CuO/NC/HMX both shifted left to the range of 38 to 44% TMD, with the same variation tendency. When AP was applied as the additive of Al/CuO, the abrupt transition zone shifted right to the range of 59.9 to 69.9% TMD, with the highest pressurization characteristics compared with those of the other Al/CuO-THEMs.

**Figure 5:**
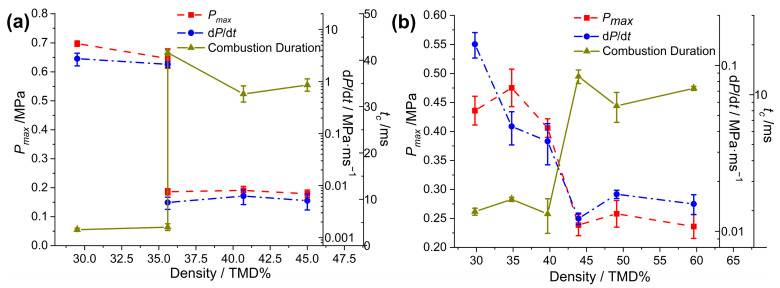
Measured pressurization characteristics of (**a**) Al/CuO/NC(10%) and (**b**) Al/CuO/NC/CL-20(PM) as a function of %TMD.

The abrupt transition events in the reactivity of the Al/CuO/NC(10%) composite prepared by electrospray and the counterpart of the Al/CuO/NC/CL-20 composite prepared by the ultrasonic mixing process were also tested, as shown in Figure 5. The formula of Al/CuO/NC(10%) was set as a comparison to Al/CuO/NC/CL-20 (or HMX, AP), in which the mass ratio of energetic additives to Al/CuO was 10%. We conducted six combustion cell tests of Al/CuO/NC (10%) loaded with 35.9% TMD, in which distinct pressurization performances were observed. Three trials exhibited rapid reactions (0.61 MPa, 1.48 MPa/ms, and 3.99 ms), whereas the other three trials exhibited sluggish reactions (0.135 MPa, 0.010 MPa/ms, and 41.6 ms). Therefore, 35.9% TMD is the abrupt transition point of Al/CuO/NC(10%), which is lower than that of Al/CuO/NC/CL-20 (or HMX, AP), which has worse reactivity. The abrupt transition in the reactivity of Al/CuO/NC/CL-20 (UM) occurred in the same regime as that of its as-sprayed counterpart. However, the values of d*P*/d*t* for Al/CuO/NC/CL-20 (UM) are one order of magnitude lower in the low-loading-density (<40% TMD) regime than in the high-loading-density (≥45% TMD) regime. Moreover, *P_max_* is one-third lower than that of Al/CuO/NC/CL-20 (ES).

### 2.4. Pressurization Characteristics of the Al/M_x_O_y_/NC/CL-20

**Figure 6:**
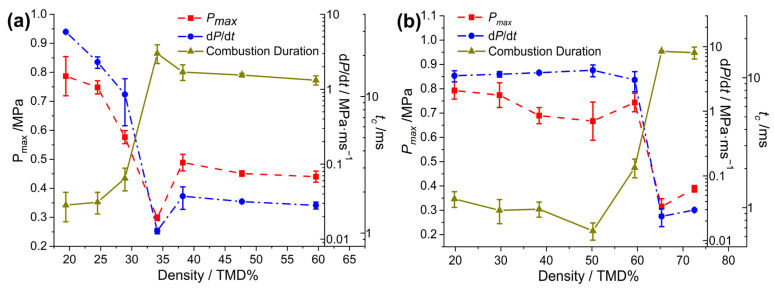
Measured pressurization characteristics of (**a**) Al/Fe_2_O_3_/NC/CL-20 and (**b**) Al/Bi_2_O_3_/NC/CL-20 as a function of %TMD.

The pressurization characteristics of Al/Oxide/NC/CL-20 at various densities are shown in Figure 6 and Table 1. When the loading density of Al/Bi_2_O_3_/NC/CL-20 varied from 59.4 to 64.3% TMD, d*P*/d*t* decreased more than two orders of magnitude from 3.05 to 0.024 MPa/ms, *P_max_* decreased from 0.74 to 0.32 MPa, and *t_c_* increased eightfold from 2.0 to 16.0 ms. For Al/Fe_2_O_3_/NC/CL-20, the abrupt transitions of pressurization characteristics appeared in the range of 28.9% to 34.1% TMD (1.07 to 1.26 g/cm^3^), during which d*P*/d*t* and *t_c_* changed by orders of magnitude. Compared to the Fe_2_O_3_- and CuO-THEMs, the Bi_2_O_3_-THEMs demonstrate superior high-speed reactivity maintenance capabilities at high loading densities.

## 3. Discussion

Once loaded nanothermites are initiated, the propagation of heat and hot, high-pressure products from the reacting zone to the unreacted zone, i.e., heat and mass transport, occurs, leading to self-sustaining reaction propagation. As estimated by Garth [28] and Kline [27], heat transfer via conduction or radiation contributes little to the energy needed to maintain the fast propagation of the reaction front. Conversely, convective heat transfer by the movement of hot gaseous and condensed products and the accompanying heat released by metal vapor condensation are the main contributors. Judging from the results, it seems that the porosity of the charge influenced by loading density determines the efficiency of convective heat and mass transfer, and the microstructure, energetic additives, and oxidant species also influence this process, leading to different abrupt transitions in reactivity.

### 3.1. Effect of Loading Density on Abrupt Reactivity Transition

For loaded nanothermites, many voids are located between reactants. These voids go against conductive heat transfer but are beneficial for convective heat transfer. The heat flux that flows from the reacting zone to the unreacted zone via conduction (*q_conduct_*) and convection (*q_convect_*) can be calculated via Equations (1) and (2) [20,44], respectively. Some assumptions that the reactive completeness of THEMs is independent of loading density, that no exothermic self-heating reaction occurs in the unreacted zone, and that the gaseous products follow the ideal gas law are proposed for simplicity. Hence, the values of *T_c_* and *C_P,g_* for each THEM at various loading densities remain constant, and *λ* and *V_g_* are variables of loading density.

(1) $$q_{conduct}=\frac{\lambda \cdot (T_{c}-T_{0})}{l}$$

(2) $$q_{convect}=\rho_{g}\cdot C_{P,g}{\cdot V}_{g}(T_{c}-T_{0})$$

where *T_c_* is the flame temperature, *T*_0_, *λ* and *l* are the initial temperature, thermal conductivity and thickness of the loaded materials, respectively, while *V_g_*, *ρ_g_* and *C_P,g_* are the velocity, density and specific heat of hot gas, respectively.

Increasing the loading density reduces the quantity of voids and enhances the interfacial contact between nanoparticles, thus improving the thermal conductivity and thermal diffusivity of the pressed pellets. However, for Al nanoparticle-based energetic materials, the increase in thermal conductivity is limited. For example, the thermal conductivities of Al [29] and Al/MoO_3_ [19] nanoparticles increase linearly from ~0.2 to ~1.0 W/m·K (much lower than that of bulk Al), with values of 1.0–2.3 g/cm^3^ and 1.07–2.52 g/cm^3^, respectively. In addition, heat transfer via conduction contributes less than 5% of the required energy; obviously, the increase in heat flux driven by conduction is insufficient to support a high propagation rate [27,28].

The voids within the loaded charges provide space for convective heat and mass transfer driven by the pressure gradient. A decrease in porosity results in a greater resistance for the transport of gaseous and condensed products. According to momentum conservation (Darcy’s law) [31,44], *V_g_* can be deduced from Equation (3), the permeability *κ* can be calculated by Equation (4) [31,44], and *x_TMD_* is defined as the ratio of *ρ_p_* to *ρ_TMD_.* Therefore, *q_convect_* can be expressed by Equation (6), in which the porosity (or the percentage of TMD) is the only variable.

(3) $$V_{g}=-\frac{\kappa }{\mu }\frac{\partial P}{\partial x}\approx -\frac{\kappa }{\mu }\frac{\left(P_{c}-P_{0}\right)}{l}$$

(4) $$\kappa =\frac{{d_{p}}^{2}\varepsilon^{3}}{150{\cdot (1-\varepsilon )}^{2}}$$

(5) $$\varepsilon =1-\frac{\rho_{p}}{\rho_{TMD}}=1-x_{TMD}$$

(6) $$q_{convect}=\frac{\rho_{g}\cdot C_{P,g}}{\mu }\frac{{d_{p}}^{2}{\left(1-x_{TMD}\right)}^{3}}{150{\cdot x_{TMD}}^{2}}\frac{\partial P}{\partial x}\left(T_{c}-T_{0}\right)$$

where *μ* is the viscosity of the hot gas, ∂*P/*∂*x* is the pore pressure gradient, *ε* is the porosity, *d_p_* is the diameter of the solid particles, *P*_0_ is the initial pressure, *P_c_* is the combustion pressure, and *ρ_p_* and *ρ_TMD_* are the loading density and TMD of the THEMs, respectively. As shown in Figure S4, the value of function y = (1 − *x_TMD_*)^3^/*x_TMD_*^2^ decreases by one order of magnitude for every 20% TMD increase, leading to a rapid decline in the convective heat flux. In comparison, the change in the convective heat transfer efficiency caused by varying porosity has a much greater effect on the transition in reactivity. Increasing the loading density compresses the free space between the particles of the THEMs and then remarkably weakens the efficiency of heat and mass transfer during self-sustaining reactions, resulting in distinct combustion performances.

### 3.2. Effect of Microstructure on Abrupt Reactivity Transition

**Figure 7:**
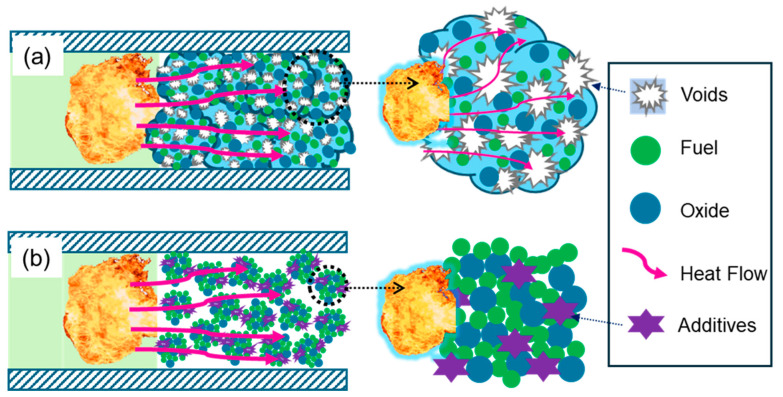
Schematics of the convective heat and mass transfer of (**a**) the electrosprayed microparticles and (**b**) their ultrasonic mixed counterparts.

Interestingly, the abrupt transition behaviors of both the Al/CuO/NC/CL-20 prepared by electrospray and that prepared by ultrasonic mixing were in the range of 40% TMD to 45% TMD. This phenomenon can be explained by the dual effects of the unique porous microstructure. The pressurization characteristics of Al/CuO/NC/CL-20 (ES) are much greater than those of Al/CuO/NC/CL-20 (UM) across all the loading densities. At low densities, the Pmax and dP/dt of Al/CuO/NC/CL-20 (ES) are more than 55% and two orders of magnitude greater, respectively. At high densities, the dP/dt values of Al/CuO/NC/CL-20 (ES) are slightly higher (18%~48%), and the margins of Pmax decrease to 30–56%. The electrospray technique has been proven to be an advanced method for assembling THEMs with superior reactivity because of the reduced distance and increased contact area between the fuel and oxide. Owing to the fast evaporation of solvents during electrospraying, a number of voids exist on the surface and in the interior of the assembled microparticles, as shown in Figure 7a. This porous microstructure can provide additional channels for convective heat transfer and the advection of condensed products, which is one of the contributions to improved reactivity. Moreover, the introduction of gas agents can prevent ultrafast sintering of nanoparticles, which negatively affects the reactivity of nanothermites. Nonetheless, interior voids can misappropriate the outer space between the assembled microparticles, decelerating the process of convective heat transfer and transport of condensed products. In contrast, the outer spaces between the components of the ultrasonic mixed powders at the same loading density are relatively abundant, as shown in Figure 7b. Finally, the competition between the positive and negative effects of the porous microstructure contributes to similar abrupt transition behaviors but with different levels of reactivity.

### 3.3. Effects of Energetic Additive Species on Abrupt Reactivity Transition

To study the effects of energetic additive species on the reactivity transition, the results of the Al/CuO-THEMs were compared. After an additional 5 wt% of organic energetic additives of HMX, CL-20, or NC was introduced into Al/CuO/NC, the loading density range of Al/CuO/NC/HMX, Al/CuO/NC/CL-20, or Al/CuO/NC(10%), in which the reactivity abruptly transitions, shifted left, whereas the Al/CuO/NC/AP shifted right.

In the former case, there is no apparent difference in the microstructures (porous and particle sizes) of the assembled mixtures. However, a higher content of organic energetic additives in the mixture would occupy more space due to the low true density, going against convective heat and mass transfer. Therefore, the loading density range of Al/CuO/NC(5 wt%) with fast reactivity is larger than that of Al/CuO(10 wt%). Compared with NC, HMX and CL-20, which have excellent energy contents, are more favorable for maintaining fast reactivity at high loading densities. Considering the similar TMD and microstructures, the intrinsic reactivity of the additives is the key factor influencing the reactivity transition behavior. Excess NC weakens the reactivity of the as-sprayed composites because of the premature dissipation of reactants from each other [10,15]. In our tests, compared with Al/CuO/NC, Al/CuO/NC(10%) generated a lower pressure with a slower d*P*/d*t*, whereas Al/CuO/NC/HMX (or CL-20) maintained comparable pressurization characteristics. Therefore, in terms of the capacity to maintain fast reactivity at high loading densities, HMX and CL-20 are superior to NC.

The latter difference may be due to the different microstructures and reaction dynamics of the Al/CuO/NC/AP. As shown in Figure 2, the Al and CuO nanoparticles were aggregated by the matrix of NC and AP, which is planar rather than spherical, so the internal voids in the assembled particles are negligible. This specific microstructure can facilitate violent combustion by avoiding the sintering of nanoparticles and providing more external channels between reactants, so Al/CuO/NC/AP can maintain higher heat and mass transfer efficiencies than the prepared spherical THEMs of Al/CuO/NC, Al/CuO/NC/CL-20, and Al/CuO/NC/HMX at the same loading densities.

**Figure 8:**
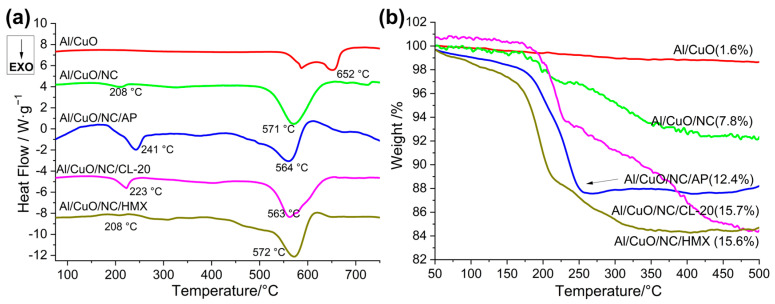
DSC (**a**) and TG (**b**) curves for the Al/CuO-THEMs.

There is a synergistic catalytic effect between the nanoparticles of Al/CuO and the energetic additives, promoting additive decomposition and thermite reaction toward lower temperatures, which can be demonstrated by the thermodynamic results [17,39]. The reaction dynamics of the Al/CuO-THEMs were investigated by a thermogravimetry–differential scanning calorimetry (TG–DSC) instrument (STA 6000, PerkinElmer, Waltham, MA, USA), as shown in Figure 8 and Figure S4. The prepared HTEMs were heated to 800 °C at a rate of 10 K/min, and argon was used as the purge gas. The TG results show that the weight loss of all the Al/CuO-THEMs exceeds the mass ratio of energetic additives, such as 7.8% for the Al/CuO/NC(5 wt%) and 12.4% or greater for the Al/CuO/NC/additives (5 wt% NC plus 5 wt% additives), leading to enhanced reaction efficiency with high flame temperature. However, the weight loss processes of the Al/CuO-THEMs are different. The weight loss of pure AP occurred between 300 and 425 °C, whereas that of the mixture counterpart occurred between 150 and 250 °C, which is much lower than the temperature of the main Al/CuO thermite reaction (500–650 °C). In contrast, the temperatures for weight loss of the other CuO-THEMs all broaden to 175–500 °C, whereas those of the pure energetic additives are below 290 °C. This means that the composite of Al/CuO/NC/AP can yield high pressure at low temperature, so the energy required for maintaining high-speed propagation during combustion is relatively low. In addition, unlike those of NC, HMX or CL-20, the decomposition products of AP containing HCl can break the alumina shell of Al nanoparticles, which is referred to as the preignition reaction (PIR), improving the solid–liquid reaction efficiency of Al and CuO [16]. These findings explain why the composite of Al/CuO/NC/AP could maintain fast combustion propagation even at a relatively high loading density.

### 3.4. Effects of Oxide Species on the Abrupt Reactivity Transition

The above results show that the loading density corresponding to the abrupt transition in Al/Bi_2_O_3_/NC/CL-20 reactivity is greater than that of the CuO- and Fe_2_O_3_-THEMs. Similar phenomena were also observed in which the abrupt transition in the propulsion performance of Al/Bi_2_O_3_/NC-2.5% occurred at 52.5% TMD, whereas that of Al/CuO occurred at 44.4% TMD [23,24]. These properties are determined by the intrinsic properties of the selected oxides, as summarized in Tables S2–S4.

Various nanothermite pairs may react via gas-phase reactions or condensed-phase reactions, leading to different combustion propagation behaviors. Under actual combustion environments or rapid heating conditions [37], the combustion initiation of Al/Fe_2_O_3_ and Al/CuO coincides with the release of gaseous oxygen from metal oxides, namely, gas-phase reactions. The combustion of Al/Bi_2_O_3_ initiates at a much lower temperature than the temperature at which oxygen is released from the bare Bi_2_O_3_, namely, the condensed phase reaction. Owing to distinct reaction mechanisms, Al/Bi_2_O_3_ ignites at a much lower temperature (850 K) than do Al/Fe_2_O_3_ (1410 K) and Al/CuO (1040 K). A lower ignition temperature indicates that a greater portion of the energy released from the loaded nanothermites is directed toward propagation rather than being consumed to initiate the unreacted materials. In addition, the oxygen release capability of oxidizers during the thermite reaction has a favorable effect on the reactivity rather than the overall thermochemistry, so Al/CuO reacts much more violently than does Al/Fe_2_O_3_ [45].

Combustion products also influence the heat transfer efficiency. Among these three nanothermite pairs, Al/CuO has the highest reaction heat, and the molecular weight of copper (63.5 g·mol^−1^) is much lower than that of Bi (209 g·mol^−1^), resulting in the highest local pressure theoretically. The boiling point and melting point of Bi are the lowest, and almost all the metal product Bi can be gasified during combustion. In contrast, only a small portion of the metal product Fe can be gasified even with high reaction heat, generating the lowest pressure. From this perspective, the upper limit of the loading density with a high propagation speed should be in the following order: Al/CuO > Al/Bi_2_O_3_ > Al/Fe_2_O_3_. However, during combustion, the low boiling point can prevent the products of Bi and Al_2_O_3_ from severely sintering, resulting in small particles [38]. This also facilitates their penetration into the pores of the unreacted zone and then releases heat due to condensation. In contrast, the Al/CuO system with large products behaves differently. In addition, the bulk density of Bi_2_O_3_ is greater; therefore, more voids exist in the loaded pellets, promoting heat and mass transfer at an identical loading density. Consequently, the Bi_2_O_3_-THEMs exhibit superior propagation rate maintenance over the CuO-THEMs at high packing densities.

## 4. Experimental Section

### 4.1. Materials

All chemical materials were purchased commercially and utilized as received. Aluminum nanoparticles (Al, 100 nm) were purchased from Haotian Nano Technology Co., Ltd. (Shanghai, China). The active content of Al was ~74.4 wt%, as determined by the TG results conducted with an air purge. Copper oxide nanoparticles (CuO, 50nm), ferric oxide nanoparticles (Fe_2_O_3_, 30 nm), and colloidal solution (4~8 wt% NC) were purchased from Aladdin Reagent Industrial Corporation (Shanghai, China). Bismuth trioxide nanoparticles (Bi_2_O_3_, 80 nm) were purchased from Beijing Deke Daojin Science and Technology Co., Ltd. (Beijing, China). Ammonium perchlorate (AP), n-hexane, ethanol and diethyl ether were purchased from Sinopharm Chemical Reagent Co., Ltd. (Shanghai, China). HMX (100 nm) and CL-20 (260 nm) nanoparticles were provided by the National Special Superfine Powder Engineering Research Center of China (Nanjing, China). SEM images of the raw materials are shown in Figure S1.

### 4.2. Sample Preparation

The equivalence ratio Φ of the nanothermites (Al/CuO, Al/Bi_2_O_3_, and Al/Fe_2_O_3_) was fixed to 1.4, which is the most intense reaction, as presented in our previous works [46]. The mass ratio of NC, HMX, CL-20, and AP was maintained at 5%. For example, the mass ratios of Al/CuO to Al/CuO/NC and Al/CuO/NC/AP are 95 wt% and 90 wt%, respectively, as shown in Table 2. A mixture of Al/CuO was prepared by a simple ultrasonic mixing procedure. The nanoparticles of Al and CuO were dispersed in n-hexane (100 mg/mL) using an ultrasonic bath for 1 h. Then, the slurry was poured into a plate to evaporate the solvent, and an ultrasonically mixed (UM) Al/CuO mixture was obtained. Nanothermites with NC and other additives, i.e., nanothermite-based hybrid energetic materials (THEMs), were assembled by an electrospray process. First, the NC and additives were dissolved in a mixture of ethanol and diethyl ether (volume ratio of 3:1) to form a homogeneous solution with a mass loading of 125 mg/mL. Then, nanoparticles of nanothermites were added to the NC solution, followed by 1 h of sonication and 24 h of magnetic agitation. The prepared precursors were transferred to a syringe and electrosprayed in a fume hood [46]. The parameters of the electrospray process were as follows: the inner diameter of the applied metal flat nozzle was 0.42 mm, the feeding rate of the precursor was 1.75 mL/h, the voltages applied on the metal nozzle and collector (flat aluminum foil) were +17 kV and −3 kV, respectively, and the distance between the nozzle and the collector was 15 cm. Finally, dry THEM powders were collected from the collector. Mechanically mixed Al/CuO/NC/CL-20 (UM) was also prepared by an ultrasonic mixing procedure as a control sample.

### 4.3. Loading Samples

To investigate the effect of loading density on the THEMs reactivities, dry powders were pressed into pellets at various densities relative to the TMD, which ranged from 20 to 80% TMD, as listed in Table 2 and Table S5. The TMD of composite materials can be calculated by Equation (7) [47], where *M_i_* and *ρ_i_* are the mass ratio and density of reactant *i*, respectively. The existence of an Al_2_O_3_ shell (~25 wt% of the Al nanoparticles) was considered for the calculation.

(7) $$TMD=\frac{1}{\sum_{i}M_{i}\frac{1}{\rho_{i}}}$$

**Figure 9:**
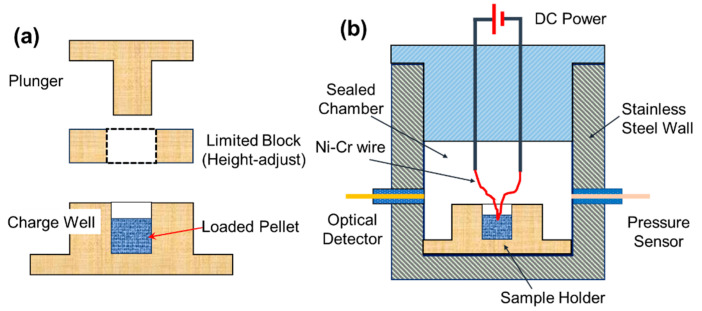
Schematics of the (**a**) pressing mold and (**b**) combustion cell.

For each test, 25.0 mg of THEMs powder was initially loaded into a cylindrical charge chamber with an inner diameter of 3.0 mm and then compressed by a top plunger, which was driven by a manual lever press, as shown in Figure 9a. Samples under various desired loading densities could be obtained by changing the height of the limit block.

### 4.4. Characterization

The morphologies of the as-prepared THEMs were observed by a scanning electron microscope (SEM, Zeiss Supra 55, Oberkochen, Germany) coupled with energy dispersive X-ray spectroscopy (EDS, Xflash 6130 Bruker, Billerica, MA, USA). The operation parameters of EDS mapping were working distance of 8.5 mm, accelerating voltage of 15 kV, and image acquisition at a resolution not less than 16k × 16k pixels. The combustion behavior under ambient air was captured by a high-speed camera (Optronis CamPerform CP70-2-M-1000, Kehl, Germany). The maximum recording rate was 33,000 fps with a resolution of 128 × 72 pixels, and the exposure time was set to 15 μs. Owing to the low flame luminance of the Bi_2_O_3_-THEMs, the applied aperture was F2.8, whereas it was F32 for the remaining THEMs.

To evaluate the pressurization characteristics of the THEMs at various loading densities, a constant volume (~15 mL) combustion cell was used to record the pressure and optical profiles as a function of time. As shown in Figure 9b, the combustion cell was equipped with a pressure sensor (Sinocera CY-YD-205, Yangzhou, China) and an optical transducer (Thorlabs DET02AFC-M, Newton, NJ, USA). The details are summarized in Tables S6 and S7. Once the packed pellet in the charge well was ignited by a heating nichrome wire (Ni-Cr dia. 0.2 mm), the histories of pressure and optical emission could be simultaneously recorded by an oscilloscope (sampling rate was 50 MSa/s, storage depth was 25 Mpts). The peak pressure (*P_max_*) is the peak value of the pressure–time profile, the pressurization rate (d*P*/d*t*) can be calculated from the initial slope of the pressure rise (from 5% *P_max_* to *P_max_*), and the combustion duration (*t_cd_*) is defined as the peak width at the half height of the optical emission profile. For each test, three trials were conducted to ensure the reliability of the data. Waring: Due to the hazardous nature of nanothermite-based energetic materials and the high-voltage risks associated with electrospray, operators should exercise extreme caution and wear complete personal protective equipment, including face shields and anti-static coats, during both preparation and characterization.

## 5. Conclusions

This study systematically investigated the abrupt transitions in reactivity of different nanothermite pairs across various loading densities and the underlying regulatory mechanisms. Increasing the loading density shifts the dominant heat transfer mode during reaction propagation from convection to conduction, leading to distinct combustion behaviors for all the prepared nanothermite-based hybrid energetic materials (THEMs). However, owing to the different porosities, microstructures, synergistic catalytic effects, and intrinsic properties of the metallic oxides, the abrupt changes in the reactivities of the Bi_2_O_3_-, Fe_2_O_3_-, and CuO-THEMs correspond to different loading density ranges. The unique porous microstructure produced by electrospray enhances the heat and mass transfer within the assembled Al/CuO/NC/CL-20 composites, leading to a much better combustion performance than that of their physically mixed counterparts. However, the presence of internal voids reduces the interstitial space between microparticles, resulting in a critical loading density for maintaining high reactivity locates in the range of 37.9 to 43.9% TMD. The temperature range corresponding to the main weight loss stage of the Al/CuO/NC/AP is much lower than that of the other CuO-THEMs, coupled with the preignition reaction; therefore, maintaining fast reactivity at the high loading density capacity of the applied additives was scaled as AP (59.9% TMD) > HMX ≈ CL-20 (37.9% TMD) > NC (35.6% TMD). Compared with those of Al/Fe_2_O_3_ and Al/CuO, the onset ignition of Al/Bi_2_O_3_ starts at a much lower temperature in the condensed phase reaction, and the sintering of products is much lower due to the low boiling point of the metal product Bi. These characteristics demonstrate that the Bi_2_O_3_-THEMs (59.7% TMD) are superior to Fe_2_O_3_- (28.9% TMD) and CuO-THEMs (37.9% TMD) in maintaining high reactivity at high density. These results can provide practical guidance for the optimal selection and precise tailoring of the reactivity of nanothermites for microscale initiators and pyrotechnics applications.

## Figures and Tables

**Table 1:** Loading density ranges and reactivities corresponding to the abrupt transition from fast (convective dominant) to slow (conductive dominant) combustion.

Materials	Loading Density Ranges of Abrupt Transition		Abrupt Changes (From Low to High Density)		
	(%TMD)	(g/cm^3^)	*P_max_* (MPa)	d*P*/d*t* (MPa/ms)	*t_c_* (ms)
Al/CuO (UM)	64.7~79.1	3.22~3.93	0.46/0.37	0.83/0.52	0.7/1.7
Al/CuO/NC	43.6~49.0	1.97~2.21	0.74/0.50	1.51/0.052	3.0/9.2
Al/CuO/NC(10%)	~35.6	~1.47	0.62/0.14	1.48/0.010	4.0/41.6
Al/CuO/NC/AP	59.9~69.9	2.53~2.95	0.78/0.62	4.76/0.238	2.2/4.2
Al/CuO/NC/HMX	38.3~44.3	1.61~1.86	0.71/0.27	2.71/0.013	3.3/21.1
Al/CuO/NC/CL-20	37.9~43.9	1.61~1.86	0.71/0.33	2.22/0.018	3.1/18.8
Al/CuO/NC/CL-20 (UM)	37.9~43.9	1.61~1.86	0.48/0.24	0.043/0.012	2.3/12.9
Al/Fe_2_O_3_/NC/CL-20	28.9~34.1	1.07~1.26	0.58/0.30	0.85/0.013	2.5/20.7
Al/Bi_2_O_3_/NC/CL-20	59.4~65.3	3.22~3.54	0.74/0.32	3.05/0.024	2.0/16.0

**Table 2:** Theoretical maximum densities and loading densities of the THEMs.

Materials	Additives	TMD (g/cm^3^)	Loading Density (%TMD)	Loading Density (g/cm^3^)
Al/CuO	/	4.97	23.7~79.1	1.18~3.93
Al/CuO/NC	5% NC	4.51	24.5~65.4	1.10~2.95
Al/CuO/NC(10%)	10% NC	4.14	25.1~71.2	1.04~2.95
Al/CuO/NC/AP	5% NC, 5% AP	4.22	24.7~76.2	1.04~3.22
Al/CuO/NC/HMX	5% NC, 5% HMX	4.20	22.8~70.2	0.95~2.95
Al/CuO/NC/CL-20 (ES and PM)	5% NC, 5% CL-20	4.24	23.8~69.5	1.01~2.95
Al/Fe_2_O_3_/NC/CL-20	5% NC, 5% CL-20	3.71	19.4~59.6	0.71~2.21
Al/Bi_2_O_3_/NC/CL-20	5% NC, 5% CL-20	5.42	19.8~72.5	1.07~3.93

## Data Availability

All the data are available on request from the corresponding authors.

## Supplementary Materials

The following supporting information can be downloaded at: https://www.mdpi.com/article/10.3390/molecules30204101/s1, Figure S1: EDS mapping of (a) Al/CuO/NC/AP, (b) Al/CuO/NC/Cl-20, (c) Al/Fe_2_O_3_/NC/Cl-20 and (d) Al/Bi_2_O_3_/NC/Cl-20; Figure S2: Combustion process of THEMs packed at low and high loading density recorded by a high-speed camera (aperture was F32, exposure time was 15 μs): Al/CuO/NC at (a-1) 30% TMD and (a-2) 50% TMD; Al/CuO/NC/HMX at (b-1) 30% TMD and (b-2) 50% TMD; Figure S3: A plot of function y = (1 − *x*_TMD_)^3^/*x*_TMD_^2^ (0.05 < *x*_TMD_ < 1); Figure S4: DSC curves of the applied energetic materials; Figure S5: SEM images of raw (a) AP, (b) CL-20, (c) HMX, (d) Al, (e) Fe_2_O_3_, (f) Bi_2_O_3_ and (g) CuO; Table S1: Properties of the applied energetic additives; Table S2: Theoretical Properties of Thermite Reactions; Table S3: Thermophysical Properties of Metal Products; Table S4: Reaction performance of the nanothermite mixtures; Table S5: Theoretical densities of ingredients; Table S6: Parameters of the DET02AFC optical transducer; Table S7: Parameters of the CY-YD-205 pressure sensor.
