# Supplementary material for: Abrupt Transition of Nanothermite Reactivity: The Roles of Loading Density, Microstructure and Ingredients
Source: Molecules. 2025 Oct 15;30(20):4101. doi: 10.3390/molecules30204101 (PMC12565809; doi:10.3390/molecules30204101)
Supplement: Supplementary file 1 [file molecules-30-04101-s001.zip › molecules-3879887-supplementary.pdf]

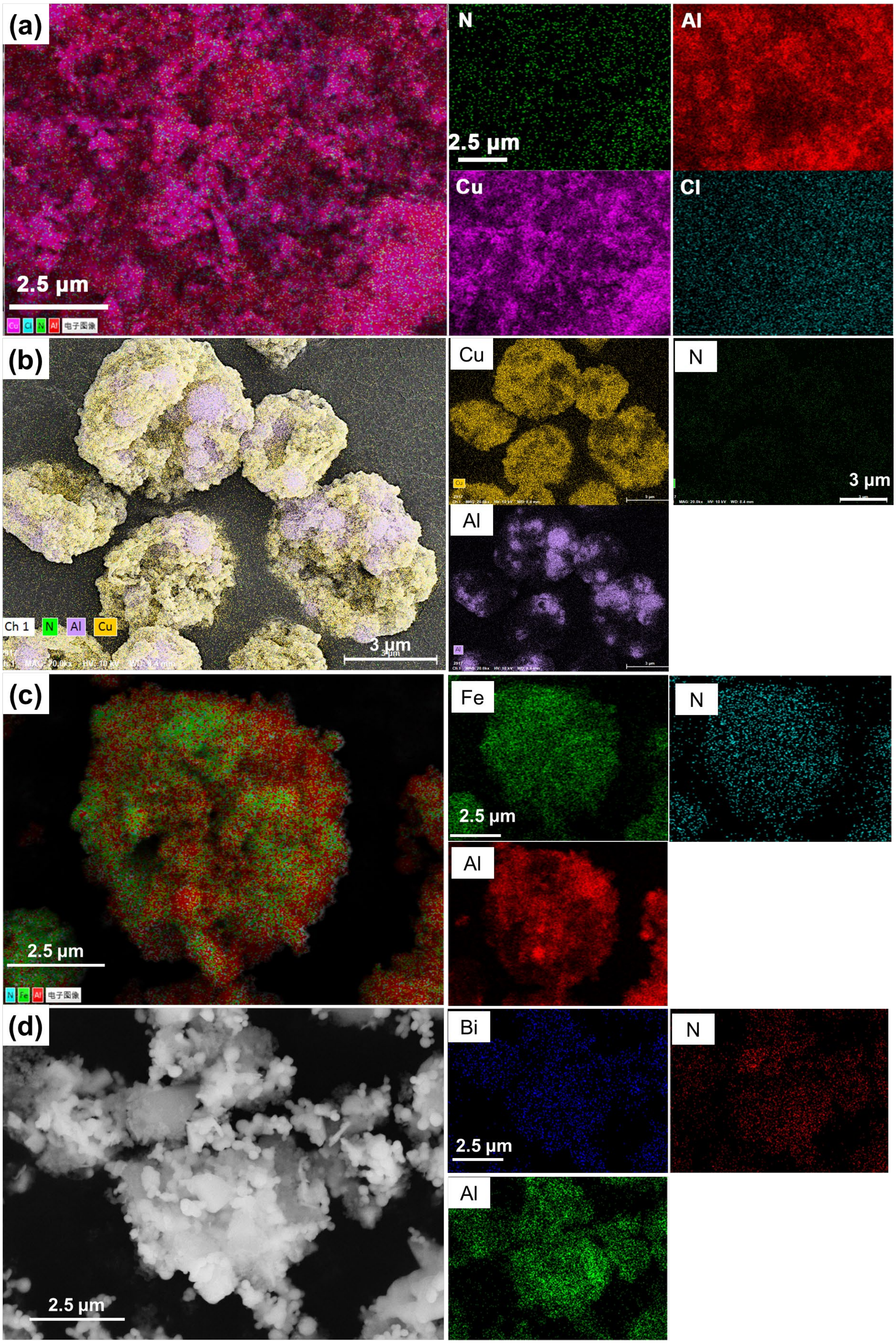

Figure S1. EDS mapping of (a) Al/CuO/NC/AP, (b) Al/CuO/NC/Cl-20, (c) Al/Fe_2O_3/NC/Cl-20 and (d) Al/Bi_2O_3/NC/Cl-20.

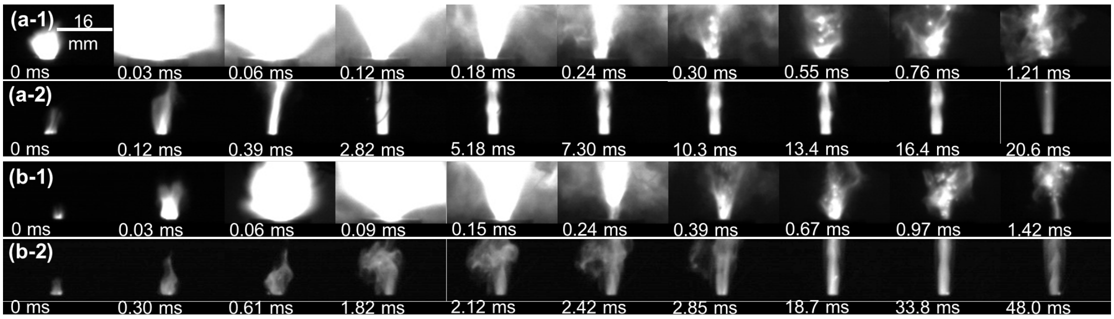

Figure S2. Combustion process of THEMs packed at low and high loading density recorded by a high-speed camera (aperture was F32, exposure time was 15 μs): Al/CuO/NC at (a-1) 30% TMD and (a-2) 50% TMD; Al/CuO/NC/HMX at (b-1) 30% TMD and (b-2) 50% TMD.

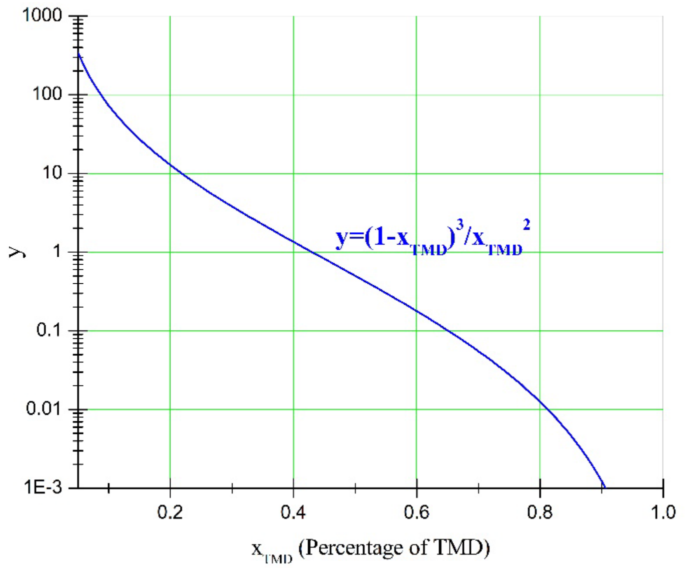

Figure S3. A plot of function y=$(1-x_{TMD})^3/x_{TMD}^2$ ($0.05<x_{TMD}<1$).

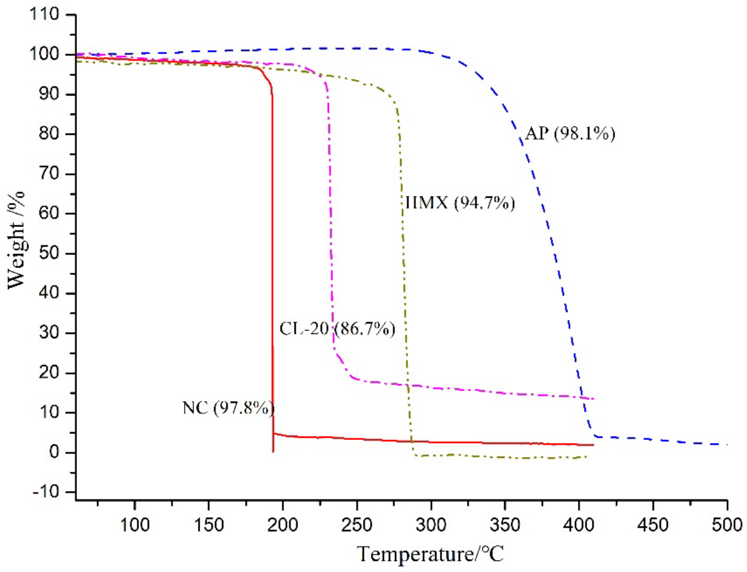

Figure S4. DSC curves of the applied energetic materials.

Table S1. Properties of the applied energetic additives.

Materials	Formula	Oxygen balance	Decomposition products
NC	$C_6H_{7.583}O_{9.835}N_{2.418}$	-35.7%	H_2O, CO, NO, CO_2, N_2, H_2
AP	NH_4ClO_4	+34.0%	N_2O, NO_2, H_2O, HCl, Cl_2
HMX	$C_4H_8N_8O_8$	-21.6%	CH_2O, HCN, NO, N_2O, H_2O, N_2, CO_2, CO
CL-20	$C_6H_6N_{12}O_{12}$	-10.1%	NO_2, NO, HCNO, HCN, CO_2

Table S2. Theoretical Properties of Thermite Reactions.

Thermites	Density (g/cm^3)	Gas Production g/g	Gas Production mol/kg	Heat of reaction J/g	Properties of Metal Products		
					State	Boiling point (K)	Melting point (K)
Al/Bi_2O_3	7.188	0.894	4.28	2118	Gas	1837	544
Al/CuO	5.109	0.343	5.40	4076	Liquid-Gas	2843	1356
Al/Fe_2O_3	4.175	0.078	1.39	3956	Liquid-Gas	3135	1811

Table S3. Thermophysical Properties of Metal Products.

Products	Density ($g{\cdot}cm^{-3}$)	Molar weight ($g{\cdot}mol^{-1}$)	Specific heat capacity at 298 K ($J{\cdot}g^{-1}{\cdot}K^{-1}$)	Specific heat capacity at 298 K($J{\cdot}mol^{-1}{\cdot}K^{-1}$)	Enthalpy of metal vaporization ($J{\cdot}g^{-1}$)
Bi	9.87	209	0.122	25.5	854.5
Cu	8.93	63.5	0.385	24.4	4812.0
Fe	7.86	56	0.449	25.1	6250.0

Table S4. Reaction performance of the nanothermite mixtures.

Nanothermites (Al + oxide)	Ignition temperature (K) (±50 K)	O_2 release temperature inthermite (K) (±50 K)	O_2 release from bare oxidizer (K) (±50 K)
Bi_2O_3	850	930	1620
CuO	1040	1050	975
Fe_2O_3	1410	1400	1340

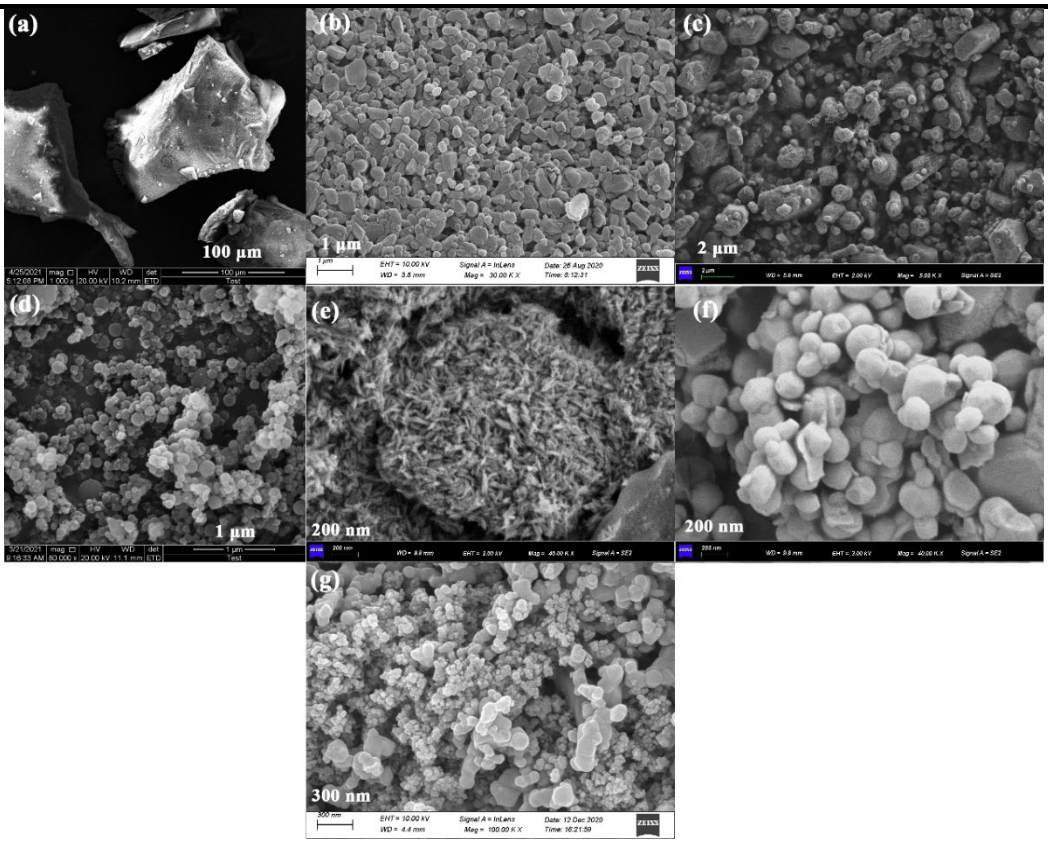

Figure S5. SEM images of raw (a) AP, (b) CL-20, (c) HMX, (d) Al, (e) Fe_2O_3, (f) Bi_2O_3 and (g) CuO.

Table S5. Theoretical densities of ingredients.

Materials	Al	Al_2O_3	CuO	Fe_2O_3	Bi_2O_3	NC	AP	HMX	CL-20
Theoretical densitiy	2.70	4.00	6.35	5.25	8.90	1.65	1.95	1.90	2.04

For the Al/CuO/NC/CL-20(5%), the mass ratio of Al, Al_2O_3, CuO, NC and CL-20 is 16.2%, 5.4%, 68.4%, 5% and 5%, respectively. So, its TMD can be calculated as:

$$TMD=1/(2.7/0.162+4/0.054+6.35/0.684+1.65/0.05+2.04/0.05)=4.24\ g/cm^3$$

Table. S6. Parameters of the DET02AFC optical transducer

Optical transducer	DET02AFC
Wavelength Range	400 - 1100 nm
Bandwidth (-3 dB)	1 GHz
Minimum Resistor Load	50 Ω
Maximum Peak Power	18 mW
Output Voltage	0 to 3.3 V (50 Ω); 0 to 10 V (Hi-Z)
Rise Time (t_r)	1 ns @ 730 nm (Max)
Fall Time (t_f)	1 ns @ 730 nm (Max)
Bias Voltage	12 V
Dark Current	126 pA
Junction Capacitance	1.73 pF (Max)
Photodiode Element	FDS02

Table. S7. Parameters of the CY-YD-205 pressure sensor.

Pressure sensitivity(20±5°C)	~ 100 pC/MPa
Measurement Range	0~60 MPa
Overload	120%
Non-linearity	<1% FS
Hysteresis	<1% FS
Repeatability	<1% FS
Isolation Resistance	>10^{13} Ω
Capacitance (1000Hz)	7 pF
Resonant Frequency	>100 kHz
Operating Temperature	-40~+150 °C
Case Material	High strength stainless steel
Mounting	M10×1
Sensing Element	Quartz
